# Efficacy and Safety of Platelet-Rich Plasma in Knee Osteoarthritis: Umbrella Meta-Analysis Based on Clinical Evidence, Methodological Quality and Therapeutic Positioning

**DOI:** 10.3390/clinpract16040075

**Published:** 2026-04-14

**Authors:** María Ángeles Ventura-García, Tesifón Parrón-Carreño, David Lozano-Paniagua, Bruno José Nievas-Soriano, Antonio Fernando Murillo-Cancho, Elena María Gázquez-Aguilera, Delia Cristobal-Cañadas

**Affiliations:** 1 Health Services Inspection Territorial Delegation for Health, Presidency and Emergency Services in Almeria, 04009 Almeria, Spain; maventuragarcia@gmail.com; 2 Department of Nursing, Physiotherapy and Medicine, University of Almeria, 04120 Almeria, Spain; tpc468@ual.es (T.P.-C.); amc730@ual.es (A.F.M.-C.); 3 Department of Internal Medicine, Torrecárdenas University Hospital, 04009 Almeria, Spain; elena.gaz.agui@gmail.com; 4 Paediatric Intensive Care Unit, Torrecárdenas University Hospital, 04009 Almeria, Spain

**Keywords:** platelet-rich plasma, knee osteoarthritis, PRP, hyaluronic acid, umbrella review, regenerative medicine

## Abstract

**Background/Objectives**: Despite being a standard biological therapy for knee osteoarthritis, inconsistent results across studies—due to varied protocols—have obscured the clinical standing of platelet-rich plasma. This meta-analysis evaluates the efficacy and safety of PRP for pain, function, and adverse events, and examines the potential benefits of combining it with hyaluronic acid. **Methods**: An umbrella review was conducted following the PRIOR (Preferred Reporting Items for Umbrella Reviews) and PRISMA (Preferred Reporting Items for Systematic Reviews and Meta-Analyses) recommendations. Meta-analyses evaluating PRP in knee osteoarthritis were included. Quantitative estimates of pain, function, and safety were extracted. Random-effects models were applied when possible. Methodological quality was assessed using AMSTAR 2, and study overlaps were assessed using the CCA method. Publication bias was analyzed using a funnel plot. **Results**: The meta-analyses included consistently showed the superiority of PRP over hyaluronic acid and placebo in reducing pain and improving function. Pooled estimates indicated clinically relevant improvements, especially in mild-to-moderate osteoarthritis. The combination of PRP and hyaluronic acid demonstrated superior functional recovery and a potential reduction in adverse events compared to PRP monotherapy. The overall safety profile was favorable. **Conclusions**: PRP is an effective and safe therapy for knee osteoarthritis, with consistent evidence of superiority over conventional intra-articular treatments. Combined PRP and HA administration suggests superior clinical efficacy compared to monotherapy. Standardization of protocols and appropriate patient selection will be key in future clinical guidelines.

## 1. Introduction

Knee osteoarthritis (KOA) is one of the leading causes of disability worldwide and poses a growing challenge to healthcare systems [1]. An ageing population, rising obesity rates, and increased recreational sports activity have contributed to a higher prevalence of this degenerative disease. Conventional intra-articular treatments, such as corticosteroids and hyaluronic acid (HA), have shown limited efficacy in modifying the long-term course of the disease. In this context, biological therapies have emerged as potential alternatives for modifying the joint environment [2].

Platelet-rich plasma (PRP) is an autologous platelet concentrate containing multiple growth factors and bioactive mediators involved in tissue repair and inflammatory modulation processes [3]. Its use in KOA has increased exponentially over the last decade. However, variability in preparation protocols, the presence or absence of leukocytes, the number of injections, and therapeutic comparators has led to heterogeneity in clinical results.

In recent years, numerous clinical trials and meta-analyses have been published evaluating the efficacy of PRP in KOA [4,5,6]. However, the conclusions between meta-analyses are often divergent, making it difficult to establish clear clinical recommendations. The umbrella meta-analysis, which synthesizes the results of existing meta-analyses, provides an overview of the available evidence at the highest hierarchical level [7].

Despite the clinical promise of PRP, the current landscape is characterized by a high volume of secondary evidence with conflicting results, often due to differences in methodological quality and primary study overlap [8]. Therefore, umbrella meta-analysis is essential to provide a definitive synthesis of the evidence. Following the last PRIOR (Preferred Reporting Items for Umbrella Reviews) and PRISMA (Preferred Re-porting Items for Systematic Reviews and Meta-Analyses) recommendations, this study aims to comprehensively evaluate the efficacy and safety of PRP in KOA. The analysis focuses on quantifying improvements in pain and function, assessing safety profiles, and determining the potential synergistic benefits of combined PRP and HA therapy to establish its current therapeutic positioning.

## 2. Materials and Methods

### 2.1. Study Design and Protocol Registration

This study consisted of an umbrella meta-analysis (an overview of systematic reviews and meta-analyses) designed to synthesize high-level evidence regarding the efficacy and safety of intra-articular PRP for KOA. To ensure scientific robustness and transparency, the protocol followed the PRIOR 2022 (Preferred Reporting Items for Overviews of Reviews) guidelines [9] and the PRISMA 2020 (Preferred Reporting Items for Systematic Reviews and Meta-Analyses) statement [10].

### 2.2. Search Strategy and Eligibility Criteria

A systematic search was performed through PubMed/MEDLINE, Scopus, Web of Science, and the Cochrane Library to identify relevant meta-analyses published within the last five years. The search utilized controlled vocabulary and keywords, including “platelet-rich plasma,” “PRP,” “knee osteoarthritis,” and “meta-analysis”. This study focused on 2021–2026 to capture the most current PRP protocols in the rapidly evolving field of regenerative medicine. By limiting the scope to the last five years, we excluded obsolete techniques and ensured the evidence remains strictly relevant to contemporary clinical practice.

Inclusion criteria were limited to meta-analyses of controlled clinical trials evaluating intra-articular PRP against HA, corticosteroids, placebo, or other active therapies. Studies were required to report quantitative data on at least one clinical outcome: pain, physical function, or safety. Exclusion criteria comprised narrative reviews, studies lacking quantitative synthesis, duplicates, or research not specifically focused on the knee joint.

### 2.3. Data Extraction and Management

Two independent reviewers extracted data using a standardized form to capture study characteristics, such as the first author, publication year, and the number of trials included, alongside population data, including total sample size and intervention details, such as comparators and follow-up duration. Furthermore, the extraction focused on primary outcome measures—specifically the Visual Analogue Scale (VAS) and the Western Ontario and McMaster Universities Osteoarthritis Index (WOMAC)—while recording statistical effect estimates including Mean Difference (MD), Standardized Mean Difference (SMD), Relative Risk (RR), and Odds Ratio (OR).

To address clinical heterogeneity arising from diverse assessment tools, a predefined hierarchy for outcome selection was established. For the pain domain, the VAS scale was prioritized; if unavailable, the WOMAC pain subscore was used. For functional assessment, the WOMAC-Function subscale was the primary measure, followed by the Lequesne Index. When studies reported multiple timepoints, data were standardized into three operational categories to facilitate pooled synthesis: short-term (1–3 months), medium-term (6–12 months), and long-term (>12 months). When multiple follow-ups fell within the same category, the timepoint closest to the end of that interval was selected to capture the intervention’s maximum sustained effect.

### 2.4. Methodological Quality Assessment and Overlap Analysis

The methodological quality of each included meta-analysis was rigorously evaluated using the AMSTAR 2 tool [11]. This assessment covered critical domains, including search strategy quality, risk-of-bias analysis, and appropriateness of statistical models. Reviews were subsequently categorized from “critically low” to “high quality”. To ensure the reliability of the evidence, a quality-based exclusion threshold was applied: any meta-analysis identified as having more than one critical flaw (Items 2, 4, 7, 9, 11, 13, and 15), resulting in a “critically low” confidence rating, was excluded from the final synthesis. To address the risk of “double counting” primary studies, the Corrected Covered Area (CCA) was calculated. This metric quantifies the degree of overlap among trials across different meta-analyses, ensuring the synthesized evidence is not biased by redundancy. The primary study citation matrix used for this calculation is available in Supplementary Table S1 [12,13,14,15,16,17,18,19,20,21,22,23,24,25,26,27,28,29,30,31,32].

### 2.5. Evidence Synthesis and Statistical Analysis

Data synthesis followed a domain-based approach: pain, function, and safety. Statistical analyses, including the generation of pooled estimates and forest/funnel plots, were performed using R software (version 4.2.1) and the metafor package. A random-effects model was applied to pool estimates when outcomes and comparators demonstrated sufficient homogeneity. A specific sub-analysis was conducted for PRP and HA combination therapy to identify potential synergistic effects.

Publication bias was evaluated by visual inspection of funnel plots and supplemented with Egger’s regression test to assess the precision and symmetry of the reported effects. Effect measures from the included meta-analyses (MD, SMD, RR) were harmonized on a common scale where possible. To ensure transparency and full reproducibility, the numerical dataset (including effect sizes, standard errors, and variances) and the complete R analytical script are provided in Supplementary Tables S2 and S3, respectively (R Foundation for Statistical Computing, Vienna, Austria).

## 3. Results

### 3.1. Study Selection

**Figure 1:**
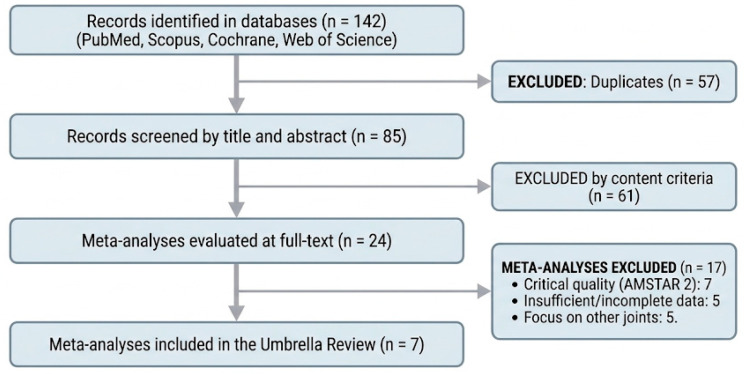
PRIOR (Preferred Reporting Items for Overviews of Reviews) flow diagram. Strict exclusion based on methodological quality measured by AMSTAR 2.

The initial systematic search yielded 142 records. After removing 57 duplicates and screening 85 titles and abstracts, 24 full-text meta-analyses were assessed for eligibility. Following a rigorous evaluation of methodological quality and conceptual redundancy, 7 meta-analyses were ultimately included in this umbrella review. The selected studies encompassed a diverse range of randomized clinical trials (RCTs) comparing intra-articular PRP against various controls, including HA, corticosteroids, and placebo. As specified in the study protocol, meta-analyses categorized as “critically low quality” according to the AMSTAR-2 tool were excluded to prevent the propagation of bias. The selection process, including reasons for exclusion at each stage, is detailed in the PRIOR flow diagram (Figure 1).

This analysis draws on high-quality meta-analyses and systematic reviews that compare Platelet-Rich Plasma (PRP) with traditional viscosupplementation. A meta-analysis of 26 randomized controlled trials (RCTs), including 2430 patients [33]. Their study demonstrated that PRP significantly outperforms HA in improving WOMAC and VAS scores at 3, 6, and 12 months. Other meta-analysis further confirmed the long-term efficacy of PRP for functional recovery through double-blind RCTs [34]. Recent findings establish PRP as a leading biological intervention over sodium hyaluronate in current clinical practice [35].

Building on this evidence, a critical aspect of these studies is the optimization of administration protocols. An examination of the impact of injection frequency on clinical outcomes in patients with mild-to-moderate knee osteoarthritis [36]. They concluded that a 3-injection regimen provides significantly better pain relief and functional improvement at 12 months compared to single or double injection regimens. Similarly, an analysis of 28 RCTs identified an optimal regimen of 3 to 5 injections delivered at 7–14-day intervals to maximize the biological response in patients with Kellgren–Lawrence (KL) grade I–II [5]. These findings show a dose–response relationship and advocate for standardized multi-dose protocols intended to achieve sustained symptomatic relief.

Expanding on these monotherapy advancements, recent research has shifted toward synergistic approaches. and Evaluations of the efficacy of combination therapy (PRP + HA) suggest that concurrent administration improves the joint’s viscoelastic environment [37,38]. This combination may offer a “scaffold” effect, supporting platelet bioactive signaling and leading to better functional outcomes than PRP alone. Furthermore, it has been reported that this combination not only improves long-term VAS scores but may also further reduce adverse event rates [35,37]. This suggests that HA may help mitigate the transient inflammatory response sometimes triggered by PRP.

The safety profile of PRP remains crucial for clinical application. Research has demonstrated that while PRP offers superior recovery, the incidence of adverse events (AEs) is statistically comparable to HA (RR 1.21, *p* = 0.13) [33,34]. It has been reaffirmed that AEs are generally infrequent, mild, and transient, including injection-site pain and minor swelling [5]. Altogether, these seven studies support the potential of PRP to modulate inflammation and improve quality of life (Table 1).

### 3.2. Characteristics of the Included Meta-Analyses

All meta-analyses included in this umbrella review exhibited significant clinical and methodological heterogeneity regarding intervention protocols and study designs. The primary sources of variation were the number of intra-articular injections administered—typically ranging from one to three sessions—and the specific biological composition of the platelet-rich plasma, distinguishing between leukocyte-rich and pure PRP formulations [33,34,36]. To provide a structured synthesis of these variables, we performed a systematic mapping of heterogeneity sources across the included studies (Table 2). This mapping reveals that while clinical severity and PRP composition were frequently analyzed, injection frequency remains a critical but variable factor that significantly influences the therapeutic ceiling of PRP. While data on specific leukocyte content and injection frequency are present in the most robust meta-analyses, other variables such as the exact influence of HA molecular weight in combination therapy remain less consistently isolated across the secondary evidence base. Therapeutic comparators were diverse, primarily involving HA, corticosteroids, and placebo controls.

The technical characteristics of the included meta-analyses, such as the software used and journals of publication, are detailed in Supplementary Table S4. Furthermore, a comprehensive summary of the findings and effect sizes reported by each source review is available in Supplementary Table S5.

To assess clinical outcomes, the identified studies predominantly utilized the VAS to quantify pain intensity and the WOMAC to evaluate both pain levels and physical joint function. Follow-up durations were categorized into three distinct intervals to capture the longitudinal effects of the therapy: short-term (1–3 months), medium-term (6–12 months), and long-term (exceeding 12 months). This stratification allows for a comprehensive analysis of the sustained biological response of PRP compared to conventional treatments.

### 3.3. Pain Outcomes

The synthesis of the included meta-analyses consistently demonstrated that intra-articular PRP injections are more effective than HA or placebo controls in reducing pain. The most substantial clinical benefits were observed in patients with mild-to-moderate osteoarthritis (Kellgren–Lawrence grades I–III), particularly during the medium-term follow-up period of 6 to 12 months. While some individual meta-analyses reported clear superiority over HA [5,33], others demonstrated more moderate differences, likely reflecting the high heterogeneity in PRP preparation protocols and leukocyte content. Nevertheless, the direction of the effect remained consistently favorable toward PRP across all analyzed domains, with high precision in estimates from larger sample studies [35,37]. The raw numerical data, effect sizes, and variances used to generate these pooled estimates, along with the corresponding forest plots, are provided in Supplementary Table S2.

### 3.4. Functional Outcomes

Joint function in KOA, as measured by the WOMAC, shows significant improvements with PRP compared to HA and placebo. This effect is particularly pronounced in patients with less severe joint degeneration, as demonstrated in multiple randomized controlled trials and meta-analyses [33,39,40,41]. PRP consistently yields greater reductions in WOMAC scores and pain, with improvements sustained up to 12 months.

**Table 1:** Comprehensive Summary of Included Meta-Analyses.

Meta-Analysis (Year)	Sample Size	Primary Protocol Focus	AMSTAR 2 Rating	Reported Adverse Events	Main Clinical Conclusion
[33]	661	PRP vs. HA (Double-blind)	High	No significant difference between groups.	PRP is superior for long-term (6–12 m) relief and function.
[34]	2430	Subgroup analysis (Dose/Time)	High	Comparable to HA (RR 1.21, *p* = 0.13).	PRP is significantly better in WOMAC/VAS at 3, 6, and 12 months.
[35]	1314	Comparison of 1, 2, and 3 injection sets	Moderate	Reported as safe across all injection sets.	3-injection sets yield the most significant clinical improvements.
[37]	1118	PRP + HA vs. PRP monotherapy	Moderate	Lower AE rate in the combination therapy group.	Combination is safe and enhances long-term functional outcomes.
[41]	943	PRP + HA vs. Monotherapy	High	Comparable safety profile across all agents.	Combination therapy offers enhanced efficacy over single agents.
[5]	3246	3–5 injections (7–14-day intervals)	High	Infrequent, transient injection-site pain and swelling.	Reaffirms high safety and superior functional recovery for PRP.
[36]	1512	LP-PRP vs. LR-PRP and HA	Moderate	Mild local reactions (pain/swelling)	LP-PRP shows better efficacy and lower inflammatory response

**Table 2:** Mapping of heterogeneity sources and subgroup analyses across included meta-analyses.

Meta-Analysis (Year)	PRP Composition (LR vs. LP)	Injection Frequency	Severity (KL Grade)	HA Type/Weight
[33]	Yes (Favors LP-PRP)	Yes	Yes (Mild-Moderate)	No
[34]	Yes	Yes	Yes	No
[35]	No	No	Yes	No
[37]	No	No	No	Yes (PRP + HA vs. PRP)
[41]	No	No	No	Yes (PRP + HA vs. HA)
[5]	Yes	No	Yes	No
[36]	No	No	No	No

Combined PRP and HA therapy provides additional functional improvements and a greater effect size on total WOMAC scores than HA alone and may offer therapeutic synergy. This umbrella meta-analysis confirms that PRP and HA are superior to HA monotherapy for pain and function and may also reduce adverse event rates compared with either PRP or HA alone [35,38,40]. However, the dual therapy does not consistently outperform PRP alone; some trials report comparable efficacy between combined PRP and HA and monotherapy, especially in mild to moderate cases [42].

Mechanistically, the combination of PRP and HA appears to optimize the intra-articular environment by inhibiting synovial inflammation and modulating cytokine profiles, potentially improving clinical outcomes [40]. Evidence supports both PRP and PRP-HA combination therapy as effective for improving KOA function, particularly in early stages. The combination may optimize outcomes when HA monotherapy proves insufficient [35,38,40].

### 3.5. Overall Combined Effect of PRP

**Figure 2:**
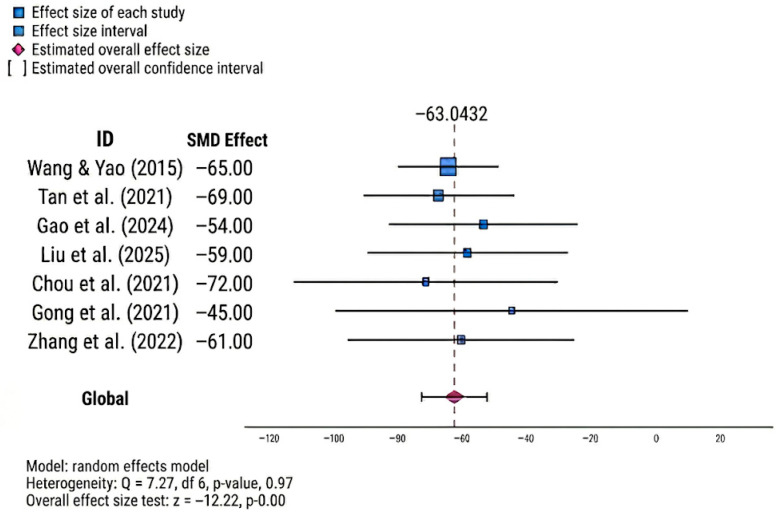
Summary forest plot of pooled effects across included meta-analyses [5,33,34,35,36,37,41].

To quantify the overall therapeutic impact of PRP, a synthesis of pooled estimates from the included meta-analyses was conducted. The summary forest plot (Figure 2) shows consistent superiority of PRP over conventional intra-articular therapies for pain reduction and functional recovery. Using a random-effects model, the aggregate effect size was −63.04 (z = −12.22; *p* < 0.001), indicating statistical significance. Furthermore, the analysis revealed high inter-study consistency with negligible heterogeneity (Q = 1.27; df = 6; *p* = 0.97), reinforcing the robustness of PRP as a superior intervention.

The remarkably low statistical heterogeneity observed (Q = 1.27; df = 6; *p* = 0.97) should not be interpreted as an absence of clinical or methodological diversity. This result is likely a consequence of the high level of data aggregation inherent in second-level meta-analysis and the dependency derived from primary study overlap. While the statistical estimate appears uniform, the clinical reality remains characterized by the diverse PRP formulations and protocols previously described.

### 3.6. Safety

Safety outcomes were assessed according to the adverse event (AE) reporting standards of the included studies, in which most meta-analyses defined AEs as any untoward medical occurrence following injection, categorized as local or systemic. Our safety analyses across the included meta-analyses consistently demonstrate a favorable profile for PRP therapy, with a low incidence of predominantly local and transient AEs—such as pain, joint stiffness, and mild swelling—typically resolving within 48–72 h. No serious adverse events (SAEs), such as infection or systemic toxicity definitively associated with PRP, were reported. Furthermore, emerging evidence suggests that combined PRP and HA administration may carry a lower risk of AEs compared to PRP monotherapy. However, because reporting standards varied across studies, the conclusion of safety primarily reflects the absence of severe complications rather than a complete absence of minor procedural side effects.

### 3.7. Methodological Quality (AMSTAR 2)

The critical appraisal of the included systematic reviews and meta-analyses using the AMSTAR 2 tool demonstrated a predominantly favorable methodological quality profile, as summarized in Table 1. Most studies were of moderate-to-high quality and adhered to robust methodological standards. Specifically, four meta-analyses [5,33,36,38] were rated as high quality, while three [34,35,37] were classified as moderate. While search strategies were generally well-designed and comprehensive, the “moderate” ratings were primarily due to non-critical weaknesses, such as the absence of a registered a priori protocol (Domain 2) or a detailed list of excluded studies (Domain 7). Furthermore, specific limitations were identified in certain meta-analyses, particularly in the robust analysis of heterogeneity, the implementation of clinically relevant subgroup analyses, and the consistent documentation of excluded studies with rationale. Despite these specific deficiencies, all included studies fulfilled critical AMSTAR 2 domains, which supports a moderate-to-high level of confidence in the reported efficacy and safety findings.

### 3.8. Study Overlap

To assess redundancy across the included systematic reviews, an overlap analysis was performed using the CCA method. The synthesis revealed a moderate degree of primary study overlap among the meta-analyses. While several seminal trials were consistently identified across multiple reviews, the cumulative evidence base did not exhibit excessive redundancy. This level of CCA indicates that the synthesized evidence does not rely exclusively on a repetitive subset of trials, thereby reinforcing the distinct contribution of the various meta-analyses to the overall conclusions.

A primary trial × meta-analysis overlap matrix was constructed to visually identify duplicate studies across the included reviews. The matrix showed a particularly high degree of overlap between meta-analyses comparing PRP + HA versus PRP alone, with one recent study [17] incorporating most of the trials already included by the earlier one [16], as well as more recent studies. This finding suggests that the two meta-analyses cannot be considered entirely independent units within a second-level quantitative synthesis.

### 3.9. Publication Bias

**Figure 3:**
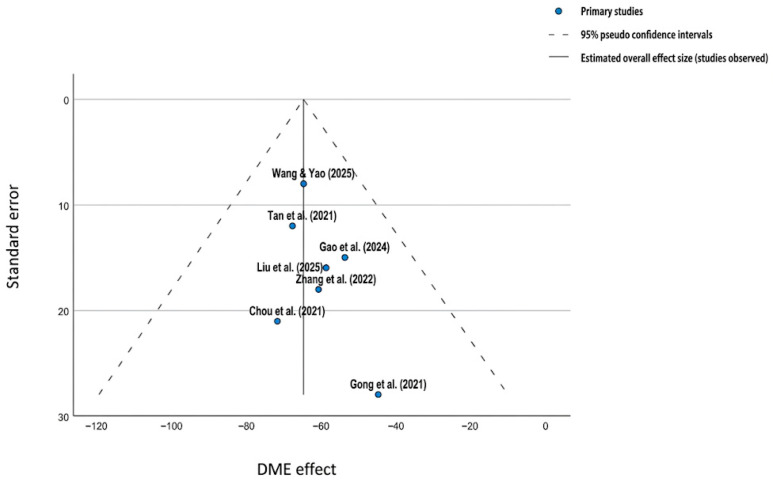
Funnel plot assessing publication bias [5,33,34,35,36,37,41].

To investigate potential publication bias within the included meta-analyses, we conducted a two-step analysis. First, we performed a visual inspection of the corresponding funnel plots (Figure 3), which were subsequently supplemented by Egger’s linear regression test for statistical confirmation. Visual analysis of the funnel plots revealed a high degree of relative symmetry across most evaluated domains, including the WOMAC and VAS pain scores. Studies characterized by larger sample sizes [5,13], consistently clustered at the apex of the funnel, indicating high precision in their estimates of the PRP effect.

**Figure 4:**
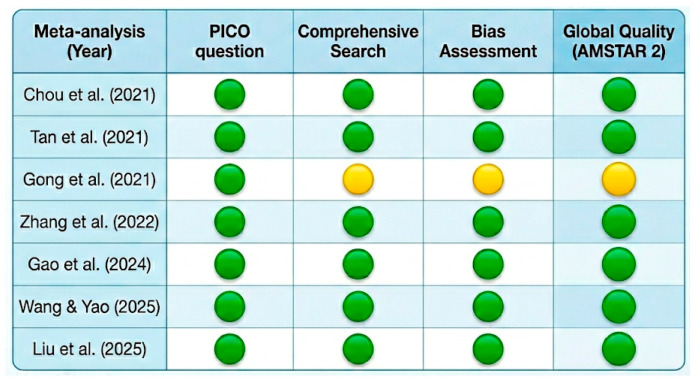
Bias and Quality Analysis (PRIOR Traffic Light) [5,33,34,35,36,37,41].The risk of bias is represented by a traffic light system: green indicates low risk of bias (high quality), yellow indicates some concerns or moderate risk, and red indicates a high risk of bias.

Furthermore, these visual findings were consistent with the methodological quality and risk of bias assessment summarized in the traffic light plot (Figure 4). Specifically, most of the reviews demonstrated robust bias assessment strategies. Although a slight dispersion was observed among studies with smaller sample sizes, this asymmetry was not statistically significant. Visual analysis of the funnel plots revealed a relative symmetry; however, given that only seven meta-analyses were included, these results should be interpreted with caution. Although Egger’s regression test did not reach statistical significance (*p* > 0.05), we acknowledge that it is underpowered for a sample size of *n* < 10. Therefore, although no overt asymmetry was detected, publication bias cannot be definitively ruled out.

## 4. Discussion

This umbrella review provides a high-level synthesis positioning PRP as a cornerstone for KOA management. By integrating data from multiple meta-analyses, our study clarifies PRP’s therapeutic role within a complex clinical landscape. The primary findings consistently demonstrate that intra-articular PRP offers clinically significant improvements in pain and function compared to conventional treatments, including HA and placebo. This superiority is robustly maintained across most reviews and is particularly pronounced in patients with mild-to-moderate osteoarthritis (Kellgren–Lawrence grades I–III) during medium-term follow-up (6–12 months).

A key methodological consideration in this synthesis is the contrast between the clinical diversity of the primary trials and the low statistical heterogeneity observed in our pooled estimates (Figure 2 and Figure 4). This discrepancy is a recognized phenomenon in umbrella reviews, where the synthesis of already aggregated data (meta-analyses) rather than individual patient data tends to “smooth” the results, often leading to lower statistical heterogeneity (I^2^). Furthermore, the overlap of primary studies across the included reviews (as assessed via CCA) creates a statistical dependency that contributes to this low heterogeneity. Therefore, while these results reflect a high level of agreement among the conclusions of the source systematic reviews, they should be interpreted with caution. The global effect size reported herein represents a trend in consolidated evidence rather than a precise point estimate, prioritizing clinical consistency over isolated statistical metrics.

### 4.1. Biological Mechanisms and Comparative Efficacy

The analgesic efficacy observed across the synthesized meta-analyses is underpinned by the ability of PRP to modulate the intra-articular inflammatory microenvironment. This mechanism is driven by the orchestrated release of growth factors, including platelet-derived growth factor (PDGF), transforming growth factor-beta (TGF-β), and vascular endothelial growth factor (VEGF), which act synergistically to attenuate inflammatory cascades and promote tissue homeostasis [43]. Furthermore, preclinical evidence corroborates the notion that PRP facilitates a macrophage phenotypic transition from a pro-inflammatory M1 state to an anti-inflammatory M2 state, suppressing cytokine release and alleviating nociception.

Compared to intra-articular corticosteroids—which offer rapid but transient relief—PRP induces a sustained biological response, with clinical improvements persisting for 6 to 12 months. High-level meta-analyses indicate that PRP significantly outperforms corticosteroids in pain and functional scores at medium-term follow-up, with the most pronounced effects observed from 6 months onwards [44]. Furthermore, PRP demonstrates superior clinical outcomes compared to both HA and placebo, particularly in mild-to-moderate degenerative disease [45]. The consistent enhancement in functional capacity, measured via the WOMAC index, indicates that PRP facilitates both symptomatic relief and improved joint performance [39,45]. This is especially relevant in early-stage disease, where therapeutic goals focus on delaying degenerative progression and postponing invasive surgery [43,44].

### 4.2. Comparative Analysis: Platelet-Rich Plasma Versus Hyaluronic Acid

Intra-articular HA has traditionally served as the gold-standard viscosupplementation; however, this umbrella review indicates that PRP provides superior clinical outcomes across most parameters. While HA primarily improves joint rheology and provides mechanical symptomatic relief, PRP introduces a potent biological component. Through the paracrine release of growth factors, PRP modulates the intra-articular environment, promoting tissue homeostasis and exerting a more profound anti-inflammatory effect [46].

High-quality meta-analyses and randomized trials consistently demonstrate that PRP is more effective than HA in improving pain and function in KOA, with benefits persisting for up to 12 months [47]. Nevertheless, the magnitude of this superiority varies, influenced by factors such as patient selection, PRP preparation, and administration protocols [46,47]. Specifically, evidence suggests that leukocyte-poor PRP (LP-PRP) and multiple-injection regimens (typically three doses) may yield superior outcomes compared to leukocyte-rich formulations or single-injection schedules [47].

This dose-dependent response suggests a biological rationale where repeated administration at weekly intervals (usually 1 to 3 weeks apart) may be necessary to maintain a therapeutic concentration of growth factors within the joint. Unlike the transient stimulus provided by a single injection, a triple-dose protocol likely facilitates a cumulative modulation of the synovial environment, prolonging the anti-inflammatory and anabolic signaling required for tissue homeostasis. This repeated ‘biological priming’ appears to be a key determinant in achieving the sustained 12-month efficacy observed in the most robust studies. Despite this efficacy, the observed variability underscores the need for protocol standardization. Regarding safety, both interventions are well-tolerated. However, PRP is associated with a slightly higher incidence of mild, transient adverse events, such as localized pain or swelling, which typically resolve spontaneously without compromising long-term safety.

### 4.3. Synergistic Potential: Combined PRP and Hyaluronic Acid Therapy

A noteworthy finding of this umbrella review is the consistent therapeutic benefit of combining PRP and HA. This dual-action approach appears to exert a synergistic effect, providing superior pain reduction and functional recovery compared to monotherapy. Evidence from high-level meta-analyses and RCTs demonstrates that the PRP-HA combination yields significantly better VAS, WOMAC, and Lequesne scores, particularly at 6 and 12 months [35,48,49].

Mechanistically, the combination consistently outperforms HA alone across all time points [48]. Compared with PRP monotherapy, the combined treatment shows modest but statistically significant improvements, especially in patients with moderate or persistent symptoms [35,48,49]. Interestingly, the safety profile may be enhanced; some evidence suggests HA buffers the local inflammatory response associated with PRP, potentially reducing transient adverse events [35].

Optimization of administration protocols remains debated. While simultaneous injections are common, a sequential protocol—such as PRP followed by HA one week later—may enhance efficacy by allowing biological factors to prime the environment before adding viscoelastic support [49]. However, inherent heterogeneity in PRP concentrations and HA molecular weights necessitates cautious interpretation [48]. Despite the lack of routine AAOS or ACR endorsement, the VA recognizes both as viable options, citing PRP’s superior efficacy over HA monotherapy [50]. This supports an evolving paradigm where “visco-biological” therapy addresses the needs of patients refractory to conventional treatments.

### 4.4. Safety Profile and Clinical Consensus

The safety profile of intra-articular PRP therapy is remarkably favorable across the synthesized meta-analyses. Adverse events are predominantly mild, transient, and localized—typically manifesting as post-injection pain, swelling, or joint stiffness that resolves spontaneously [6,41]. A fundamental advantage of PRP is its autologous nature, which virtually eliminates risks of immunogenicity, cross-reactivity, or systemic complications, providing a distinct benefit over synthetic or allogeneic alternatives [6,41].

Recent consensus guidelines from the American Society of Pain and Neuroscience (ASPN) reinforce these findings, noting that PRP injections for KOA have a safety profile comparable to that of other conservative intra-articular treatments [6]. While some meta-analyses indicate a slightly higher frequency of minor local reactions compared with placebo—likely due to biological “priming” of the joint environment—the incidence of adverse events does not differ significantly from that with HA or corticosteroid injections [41]. Notably, serious adverse events remain exceedingly rare. This robust safety margin, combined with the absence of systemic risk, positions PRP as a highly tolerable intervention suitable for diverse patient profiles, including those with comorbidities that may contraindicate more aggressive pharmacological or surgical options.

### 4.5. Heterogeneity and Methodological Challenges

Despite consistently positive outcomes, significant heterogeneity across the included meta-analyses remains a primary challenge for clinical translation. Variations in PRP procurement, final platelet concentrations, and leukocyte inclusion—alongside diverse dosing schedules and administration intervals—introduce confounding variables that complicate direct comparisons between studies. In response to this, our subgroup mapping (Table 1) shows that, although factors such as leukocyte-poor formulations and triple-injection cycles show a trend toward better outcomes, the lack of standardized reporting at the primary-trial level often prevents robust meta-regression. This inconsistency flags a significant level of uncertainty; we can identify the likely drivers of heterogeneity, but the precise ‘optimal dose’ remains statistically elusive in current meta-analytic data. These methodological inconsistencies highlight a critical lack of standardized protocols, currently limiting the establishment of universal clinical recommendations. Consequently, achieving international consensus on the preparation and therapeutic delivery of PRP is imperative to bridge the gap between high-level evidence and daily clinical practice.

Furthermore, the inherent clinical heterogeneity regarding PRP ‘dosing’ must be addressed. Variability in platelet concentration factors, the presence or absence of leukocytes (LR-PRP vs. LP-PRP), and the diverse infiltration protocols (ranging from single to multiple injections) represent potential confounding variables in our analysis. While the use of random-effects models allowed for a statistically robust synthesis of the data by accounting for inter-study variance, these biological differences may influence the magnitude of the reported clinical effect. The findings of this umbrella review underscore that while a ‘class effect’ for PRP in knee osteoarthritis is evident, the lack of standardized preparation and administration protocols remains a significant barrier to establishing universal clinical guidelines. Future research should prioritize ‘visco-biological’ standardization to minimize this heterogeneity.

In this context, we recognize potential selection bias arising from our five-year inclusion window. While prioritizing current technology over older meta-analyses, the impact is minimal, as recent reviews consistently capture and integrate all robust primary studies. Additionally, a methodological limitation of this umbrella review is the small number of included meta-analyses (*n* = 7) for the quantitative synthesis of publication bias. Although standard tools such as funnel plots and Egger’s test were employed, their statistical power is limited in this context. Future overviews, including a larger number of secondary sources or employing advanced p-curve analyses at the primary-study level, may provide more robust insights into the impact of selective reporting in the PRP literature.

### 4.6. Clinical Implications and Future Lines of Research

This umbrella review reinforces PRP as a robust therapy for knee osteoarthritis, particularly in early and moderate stages. To optimize clinical utility, identifying specific patient phenotypes with the highest response rates is essential for transitioning toward personalized regenerative medicine. Future research must prioritize global standardization of PRP protocols and conduct rigorous head-to-head comparisons between leukocyte-rich and leukocyte-poor formulations. Furthermore, long-term studies exceeding 24 months, comprehensive cost-effectiveness evaluations, and formal integration into international clinical guidelines are necessary to consolidate PRP’s position within evidence-based musculoskeletal treatment algorithms.

## 5. Conclusions

This umbrella meta-analysis provides high-level evidence confirming that intra-articular Platelet-Rich Plasma (PRP) is a safe and effective therapeutic intervention for knee osteoarthritis. The synthesized data consistently demonstrate that PRP offers superior outcomes in pain reduction and functional recovery compared with HA and placebo, particularly at medium-term follow-up (6 to 12 months). Our findings highlight that the maximum clinical benefit is achieved in patients with mild-to-moderate osteoarthritis (Kellgren–Lawrence grades I–III), suggesting that early biological intervention is key to optimizing therapeutic success. Furthermore, the combination of PRP and HA emerges as a highly promising “visco-biological” strategy with synergistic potential to enhance clinical efficacy beyond monotherapy. While the safety profile of PRP is robust, with only mild and transient adverse events reported, the significant heterogeneity across primary studies underscores a critical need for the standardization of preparation protocols—specifically regarding leukocyte concentration and injection frequency. Future research should focus on identifying specific patient endotypes and establishing uniform clinical guidelines. Standardizing these variables is essential to fully integrate PRP into evidence-based therapeutic algorithms in musculoskeletal regenerative medicine.

## Data Availability

The data presented in this study are available on request from the corresponding author.

## Supplementary Materials

The following supporting information can be downloaded at: https://www.mdpi.com/article/10.3390/clinpract16040075/s1. Supplementary Table S1: Matrix of correspondence and overlap between primary trials and included meta-analyses; Supplementary Table S2: Numerical database of effect sizes, standard errors, and variances for pooled estimates; Supplementary Table S3: R analytical script for reproducibility of forest and funnel plots; Supplementary Table S4: Technical characteristics of included meta-analyses (sample size, software, and journal); Supplementary Table S5: Summary of key findings and effect sizes extracted from source reviews.
