# Supplementary material for: Efficacy and Safety of Platelet-Rich Plasma in Knee Osteoarthritis: Umbrella Meta-Analysis Based on Clinical Evidence, Methodological Quality and Therapeutic Positioning
Source: Clin Pract. 2026 Apr 14;16(4):75. doi: 10.3390/clinpract16040075 (PMC13115077; doi:10.3390/clinpract16040075)
Supplement: Supplementary file 1 [file clinpract-16-00075-s001.zip › clinpract-4208503-supplementary.pdf]

Supplementary Table S1. Matrix of correspondence and overlap between primary trials and included meta-analyses.

Primary Trial	[34]	[35]	[33]	[37]	[36]	[41]	[5]
**[12]**	0	1	0	1	1	1	0
**[13]**	0	1	0	1	1	0	0
**[14]**	1	0	0	1	1	1	0
**[15]**	0	0	0	1	1	1	1
**[16]**	0	0	0	1	1	1	0
**[17]**	0	0	0	0	1	1	1
**[18]**	0	0	0	0	1	0	0
**[19]**	0	0	0	1	1	0	0
**[20]**	0	0	0	1	1	0	0
**[21]**	1	0	0	1	1	0	1
**[22]**	0	0	0	1	1	0	0
**[23]**	0	0	0	1	1	0	0
**[24]**	0	0	0	1	1	0	1
**[25]**	1	1	0	0	0	0	1
**[26]**	1	1	0	0	0	0	0
**[27]**	1	1	0	0	0	0	0
**[28]**	1	1	0	0	0	0	0
**[29]**	1	0	0	0	0	0	0
**[30]**	1	0	0	0	0	0	0
**[31]**	1	0	0	0	0	0	0
**[32]**	1	0	0	0	0	0	0

1: explicit inclusion identified; 0: not listed in the extracted material.

Supplementary Table S2. Numerical database of effect sizes, standard errors, and variances for pooled estimates.

Review	Comparison	Metric	Effect	SE	Variance	95% CI Lower	95% CI Upper
**[5]**	PRP in KOA	SMD	-63.2	8.1	65.61	-79.08	-47.32
**[34]**	PRP vs HA	SMD	-66.4	12.1	146.41	-90.12	-42.68
**[41]**	PRP+HA vs monotherapy	SMD	-51.7	15.2	231.04	-81.49	-21.91
**[36]**	PRP/HA	SMD	-57.0	16.1	259.21	-88.56	-25.44
**[37]**	PRP+HA vs PRP alone	SMD	-59.1	18.2	331.24	-94.77	-23.43
**[5]**	PRP vs HA	SMD	-42.5	28.3	800.89	-97.97	12.97
**[33]**	Number of PRP injections	SMD	-70.6	21.2	449.44	-112.15	-29.05

SMD: *Standardized Mean Difference*; **PRP / HA:** *Platelet-Rich Plasma and Hyaluronic Acid*; **SE:** *Standard Error*; **95% CI:** *95% Confidence Interval*

Supplementary Table S3. R analytical script for reproducibility of forest and funnel plots.

```
# Script de R
# Reproduction script for umbrella-level pooled estimate and Figures 2-4

# install.packages("metafor")
library(metafor)

dat <- data.frame(
  review = c("Wang & Yao (2025)",
         "Tan et al. (2021)",
         "Gao et al. (2024)",
         "Liu et al. (2025)",
         "Zhang et al. (2022)",
         "Gong et al. (2021)",
         "Chou & Shih (2021)"),
  yi  = c(-63.2, -66.4, -51.7, -57.0, -59.1, -42.5, -70.6),
  sei = c(  8.1,  12.1,  15.2,  16.1,  18.2,  28.3,  21.2)
)

dat$vi <- dat$sei^2

# Random-effects model
res <- rma(yi = yi, sei = sei, data = dat, method = "REML")
print(res)

# Forest plot
png("Figure2_forest_plot.png", width = 1800, height = 1200, res = 200)
forest(res,
      slab = dat$review,
      xlab = "DME effect",
      mlab = "Random-effects model",
      cex = 0.9)
dev.off()
```

```r
# Funnel plot
png("Figure3_funnel_plot.png", width = 1600, height = 1200, res = 200)
funnel(res,
    xlab = "DME effect",
    ylab = "Standard error",
    main = "Funnel plot")
text(dat$yi, dat$sei, labels = dat$review, pos = 4, cex = 0.7)
dev.off()

# Influence diagnostics
infl <- influence(res)
print(infl)

# Egger test (interpret with caution given small number of reviews)
egger <- regtest(res, model = "rma")
print(egger)

# Export analysis table
write.csv(dat, "S1_dataset_used_in_R.csv", row.names = FALSE)
capture.output(summary(res), file = "meta_summary.txt")
```

Supplementary Table S4. Technical characteristics of included meta-analyses (sample size, software, and journal).

Review	Clinical Question	No. of Studies	Sample Size	Software	Journal
**[34]**	PRP vs. HA in KOA	26 RCTs	2,430	Review Manager Database	Arthroscopy
**[35]**	PRP vs. HA in KOA	6 DB-RCTs	661	Review Manager 5.3	Medicine
**[33]**	Number of PRP injections in KOA	31 articles	1,224	RevMan 5.4	International Journal of Clinical Practice
**[37]**	PRP+HA vs. PRP alone	13 studies	1,118	RevMan 5.3	J Orthop Surg Res
**[41]**	PRP+HA vs. monotherapy	10 RCTs	943	Fixed/random effects model; statistical analysis reported	Arch Orthop Trauma Surg
**[36]**	PRP/HA for OA (pooled dataset reported in text)	16 studies	1,384	Meta-analysis in PLOS ONE	PLOS ONE
**[5]**	PRP for KOA vs. multiple comparators	28 RCTs	3,246	RevMan 5.4.1 / R 4.2.1 / Python 3.9 / STATA 17	Eur J Med Res

KOA: *Knee Osteoarthritis*; **RCTs:** *Randomized Controlled Trials*; **DB-RCTs:** *Double-Blind Randomized Controlled Trials*.

Supplementary Table S5. Summary of key findings and effect sizes extracted from source reviews.

Review	Clinical Question	No. of Studies	Sample Size	Software	Journal
[34]	PRP vs. HA in KOA	26 RCTs	2,430	Review Manager Database	Arthroscopy
[35]	PRP vs. HA in KOA	6 DB-RCTs	661	Review Manager 5.3	Medicine
[33]	Number of PRP injections in KOA	31 articles	1,224	RevMan 5.4	International Journal of Clinical Practice
[37]	PRP+HA vs. PRP alone	13 studies	1,118	RevMan 5.3	J Orthop Surg Res
[41]	PRP+HA vs. monotherapy	10 RCTs	943	Fixed/random effects model; statistical analysis reported	Arch Orthop Trauma Surg
[36]	PRP/HA for OA (pooled dataset reported in text)	16 studies	1,384	Meta-analysis in PLOS ONE	PLOS ONE
[5]	PRP for KOA vs. multiple comparators	28 RCTs	3,246	RevMan 5.4.1 / R 4.2.1 / Python 3.9 / STATA 17	Eur J Med Res

KOA: *Knee Osteoarthritis*; **RCTs:** *Randomized Controlled Trials*; **DB-RCTs:** *Double-Blind Randomized Controlled Trials.*
